# Increasing BMI is associated with a progressive reduction in physical quality of life among overweight middle-aged men

**DOI:** 10.1038/srep03677

**Published:** 2014-01-14

**Authors:** José G. B. Derraik, Martin de Bock, Paul L. Hofman, Wayne S. Cutfield

**Affiliations:** 1 Liggins Institute, University of Auckland, Auckland, New Zealand; 2 Gravida: National Centre for Growth and Development, Auckland, New Zealand

## Abstract

We assessed whether increasing body mass index (BMI) affects health-related quality of life in a group of 38 overweight (BMI 25–30 kg/m^2^) middle-aged (45.9 ± 5.4 years) men, recruited in Auckland (New Zealand). Health-related quality of life was assessed with SF-36v2 at 0, 12, and 30 weeks. Increasing BMI was associated with a progressive reduction in physical component summary score (p = 0.008), as well as lower general health (p = 0.036), physical functioning (p = 0.024), and bodily pain (p = 0.030) scores. Stratified analyses confirmed these findings: participants who were more overweight (n = 19; BMI 27.5–30 kg/m^2^) had poorer physical component summary (p = 0.005), physical functioning (p = 0.040), bodily pain (p = 0.044), and general health (p = 0.073) scores than the less overweight (n = 19; BMI 25–27.5 kg/m^2^). Increasing BMI is associated with a progressive reduction in physical quality of life, even within a relatively narrow BMI range encompassing only overweight middle-aged men.

There is a global obesity pandemic, and obesity rates in New Zealand continue to increase in both adults and children^1^. In 2011–2012, 28% of New Zealand's adult population were obese, compared to 19% in 1997^1^. Importantly, a further 35% of the adult population were overweight but not obese^1^. While obesity rates are identical in both sexes, men are more likely to be overweight than women (41.3 vs 32.8%)^2^.

There is ever increasing awareness of the adverse effects of obesity on morbidity and mortality, particularly via cardiovascular diseases. There is also increasing evidence that obesity affects health-related quality of life (HRQL), especially physical functioning^3^. Numerous studies have examined the effects of body mass index (BMI) on HRQL, showing that higher degrees of obesity are associated with increased burden of disease^3^,^4^,^5^,^6^,^7^,^8^. These studies demonstrated that the more obese a person is, the poorer the physical health, reflected for example in impaired ability to perform daily physical tasks.

However, the data on overweight (BMI 25–30 kg/m^2^) as compared to obesity (≥30 kg/m^2^) have yielded inconsistent findings, and some studies have not observed adverse effects on HRQL^4^,^6^,^7^,^8^. In light of the conflicting data in this particular group, we assessed whether increasing BMI affects health-related quality of life in a group of overweight middle-aged men.

## Results

### Study cohort

45 participants took part in the clinical trial^9^, but 7 were excluded due to incomplete HQRL data. Thus, 38 overweight men (BMI 27.3 ± 1.4 kg/m^2^), aged 45.9 ± 5.4 years (range 34.5–55.6) were studied. Most were of New Zealand European ethnicity (89%). Three participants were on antihypertensive medication, three were on lipid-lowering medications, and two participants were on both. No participants had any other active physical or mental health co-morbidities. Note that the trial interventions had no effect on any HRQL scores.

Compared to New Zealand normative data, study participants displayed similar scores in the mental health domains, namely vitality (p = 0.36), social functioning (p = 0.29), role emotional (p = 0.31), and mental health (p = 0.10). However, study participants displayed better physical well-being than the normative data, with higher role physical (95.5 vs 85.7; p < 0.001), physical functioning (94.5 vs 85.9; p < 0.001), bodily pain (82.5 vs 75.3; p = 0.012), and general health (80.2 vs 74.5; p = 0.009) scores.

### BMI vs HRQL

Univariate analyses showed that BMI was negatively correlated with physical component summary (r = −0.43; p = 0.007), physical functioning (ρ = −0.34; p = 0.035), and bodily pain (ρ = −0.34; p = 0.037) scores, also tending towards a similar association with general health (r = −0.30; p = 0.065) and vitality (r = −0.29; p = 0.081) scores. These results were corroborated by multivariate models, which showed that greater BMI was associated with poorer physical quality of life. Thus, increasing BMI was associated with a progressive reduction in physical component summary score (p = 0.008), as well as lower general health (p = 0.036), physical functioning (p = 0.024), and bodily pain (p = 0.030) scores.

The stratified analyses confirmed the findings that increasing BMI among overweight men was associated with a progressive reduction in physical quality of life. Participants who were more overweight (n = 19; BMI 27.5–30 kg/m^2^) had poorer physical quality of life than those were less overweight (n = 19; BMI 25–27.5 kg/m^2^) (Table 1). This was illustrated by differences in the physical component summary score (p = 0.005), as well as physical functioning (p = 0.040), bodily pain (p = 0.044), and general health (p = 0.073) scores (Table 1). There were no differences between groups in mental health domains.

## Discussion

This study shows that increasing BMI is associated with a progressive reduction in physical quality of life among middle-aged men. Importantly, this was observed to occur within a relatively narrow BMI range, encompassing only overweight men. Further, our findings were observed in an otherwise healthy group of individuals, with the possible confounding effects of physical activity levels and significant comorbidities accounted for.

The increased morbidity and mortality associated with obesity is widely recognized. However, recent studies have suggested that this is not the case for those who are overweight when compared to normal-weight individuals^13^,^14^,^15^,^16^. Similarly, the HRQL literature on the effects of overweight is conflicting, and some studies found no effects of overweight on HRQL in comparison to those of normal weight^6^,^7^,^8^. However, irrespective of whether HRQL in a group of overweight males actually differs to that of normal-weight controls, our data do show that increasing BMI is associated with poorer outcomes in physical domains, even within a group consisting solely of overweight individuals.

We did not observe any effects of BMI on mental health domains assessed. These findings are consistent with a number of previous studies showing that physical quality of life is far more affected by increasing BMI than mental health domains^6^,^17^,^18^. However, the observed reduction in physical functioning in our overweight cohort may lead to decreased energy expenditure, resulting in increasing mismatch in energy balance and additional weight gain^19^. Thus, the observed impairments in physical health in overweight individuals may eventually lead to the onset of obesity. As a result, while we did not observe any effects of increasing BMI on mental health domains, progression to obesity would likely result in adverse effects on mental health^20^.

The main limitation of our study was our relatively small number of participants (n = 38). However, our findings are particularly robust as each participant underwent three assessments over a 30-week period. Thus, our study design minimized the potential effects of temporal variations in HRQL that might have been experienced by individual participants. Nonetheless, we studied a relatively narrow range of individuals (overweight males living in a large urban centre, mostly of New Zealand European ethnicity), which may limit wider applicability of our findings.

In summary, we found that physical quality of life is affected by increasing overweight levels, in the absence of overt obesity. There is considerable focus on public health interventions targeting those who are obese. Our study shows that overweight non-obese individuals also display impairments in physical quality of life. It is important therefore that such aspects are considered in the health management of overweight individuals, not only to improve quality of life, but also to prevent further reductions in physical activity levels and halt a consequent progression to obesity.

## Methods

This study encompassed the *post hoc* analysis of data from a 30-week randomized, double-blinded, placebo-controlled, crossover trial examining the effects of olive leaf extract on insulin sensitivity^9^. All participants were overweight men (BMI 25–30 kg/m^2^) aged 35–55 years, who were recruited in February 2011 via advertisements in local newspapers that circulate freely in the central Auckland metropolitan area (New Zealand). Note that only males were recruited to the clinical trial so that the effects of the menstrual cycle on insulin sensitivity could be avoided. Exclusion criteria were: drug use (including tobacco), diabetes, or being on medications likely to affect insulin sensitivity. Subjects taking antihypertensive or lipid-lowering medications were recruited, but were required to have been on a stable dose for at least 6 months prior to start of the study. These subjects were also encouraged not to change dose throughout the trial, and doses were checked at each assessment. All participants were asked not to make any substantial alterations to their lifestyle for the duration of the trial. Specifically, participants were instructed not to make changes to their diet and physical activity levels.

**Figure 1:**
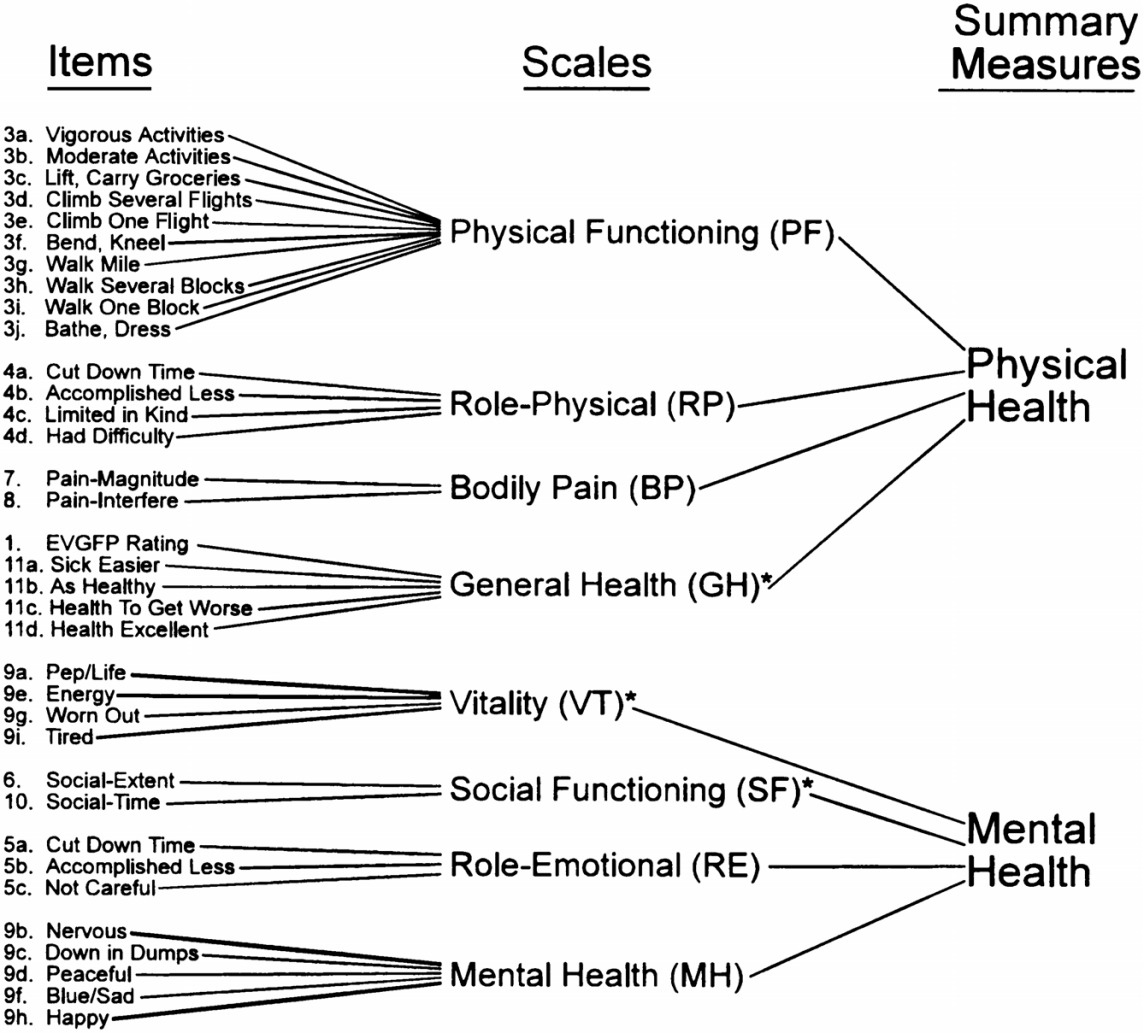
Itemized description of health-related quality of life (HRQL) measures as per the SF-36 survey. * indicates significant correlation with other summary measure. Reproduced with permission from Ware JE, Jr. SF-36® Health Survey Update; http://www.sf-36.org/tools/sf36.shtml.

Participants were assessed at baseline and after 12 and 30 weeks. Clinical assessments were carried out at the Maurice & Agnes Paykel Clinical Research Unit (Liggins Institute, University of Auckland). HRQL was assessed using the SF-36v2 Health Survey (New Zealand/Australia adaptation), based on subjective measures of well-being. The SF-36v2 is a validated tool that measures perception of health on eight multi-item dimensions covering functional status, wellbeing, and overall evaluation of health^10^. The SF-36v2 assesses 8 domains, including 4 physical (general health, physical functioning, bodily pain, and role limitations related to physical problems) and 4 mental (mental health, vitality, social functioning, and role limitations related to emotional problems)^10^. Parameters summarizing both physical and mental domains were also obtained. There are a number of items that make up each individual domain. These have been presented and discussed by Ward^10^, and are summarized in Figure 1.

Auxological assessment included height measurement using a Harpenden stadiometer. Weight was measured using whole-body dual-energy X-ray absorptiometry (DXA, Lunar Prodigy 2000, General Electric, Madison, USA). Physical activity levels were assessed using the International Physical Activity Questionnaire (IPAQ)^11^, covering four domains of physical activity: work-related, transportation, housework/gardening, and leisure time.

Univariate analyses were initially performed using simple correlations or Spearman's rank correlations. SF-36v2 data for the study cohort were compared to normative data for New Zealand men aged 45–64 years^12^ using two-sample t-tests. Random effect mixed models with repeated measures were used to evaluate the association of BMI with SF-36v2 outcomes. Randomization sequences, time period, and on-going use of medication (for cholesterol or hypertension) were accounted for, with IPAQ score also included as a co-variate. Stratified analyses were also carried out dividing the study cohort in half, separating participants according to BMI. Univariate analyses were carried out in Minitab v.16 (Pennsylvania State University, State College, PA, USA), while multivariate analyses were performed in SAS v.9.3 (SAS Institute Inc. Cary, NC, USA). Demographic data are presented as means ± standard deviation, while outcome data are presented as model-adjusted means (estimated marginal means adjusted for the confounding factors in the models), with associated 95% confidence intervals. All statistical tests were two-sided and a 5% significance level maintained throughout the analyses.

Ethics approval for this study was provided by the Northern Y Regional Ethics Committee (New Zealand Ministry of Health), and written informed consent was obtained from all participants. This study was registered with the Australian New Zealand Clinical Trials Registry (#336317).

## Author Contributions

M.d.B., W.S.C., P.L.H. and J.G.B.D. conceived and designed the experiment. M.d.B. performed the experiment. J.G.B.D. analysed the data. J.G.B.D. and M.d.B. wrote the manuscript with input from W.S.C. and P.L.H.

## Figures and Tables

**Table 1:** Health-related quality of life data on 38 middle-aged overweight men evaluated three times over a 30-week period. Demographic data are means ± standard deviations; all other data are adjusted means from multivariate models and respective 95% confidence intervals. Higher physical and mental health scores represent better outcomes

	Less overweight (BMI 25–27.5 kg/m^2^)	More overweight (BMI 27.5–30 kg/m^2^)	p-value
**n**	19	19	
**Demography at baseline**			
Age (years)	46.5 ± 5.0	45.3 ± 5.9	0.50
BMI (kg/m^2^)	26.2 ± 0.8	28.5 ± 0.7	<0.0001
Total body fat (%)	26.0 ± 3.4	31.6 ± 4.7	<0.0001
Exercise (IPAQ)	3382 ± 4359	3151 ± 3528	0.76
**Physical health**			
Physical component summary	58.4 (56.6–60.2)	55.6 (54.0–57.2)	0.005
General health	80.7 (74.9–86.5)	73.9 (68.2–79.6)	0.073
Physical functioning	95.4 (92.4–98.4)	92.0 (89.2–94.8)	0.040
Role limitations due to physical problems	95.8 (93.2–98.3)	95.2 (92.8–97.7)	0.74
Bodily pain	86.1 (79.4–92.7)	77.5 (71.1–84.0)	0.044
**Mental health**			
Mental component summary	52.5 (49.4–55.7)	51.5 (48.4–54.6)	0.58
Mental health	78.3 (72.8–83.9)	77.4 (72.0–82.7)	0.78
Vitality	67.8 (61.4–74.2)	62.7 (56.4–68.9)	0.21
Social functioning	88.7 (81.7–95.7)	92.4 (85.6–99.3)	0.39
Role limitations due to emotional problems	91.8 (86.4–97.1)	91.0 (85.7–96.2)	0.81
